# Ectopic Thyroid Tissue in the Anterior Mediastinum: A Case Report

**DOI:** 10.7759/cureus.63293

**Published:** 2024-06-27

**Authors:** Ahmed Elashmawy, Angie Achkar, Angelo Federico, John Hilu

**Affiliations:** 1 Pediatrics, Wayne State University School of Medicine, Detroit, USA; 2 Surgery, Wayne State University School of Medicine, Detroit, USA; 3 Surgery, Corewell Health East, Dearborn, USA

**Keywords:** thoracic radiology, anatomical aberrations, anterior mediastinal mass, anterior mediastinum, ectopic thyroid tissue

## Abstract

Ectopic thyroid tissue (ETT) is a rare finding on mediastinoscopy which could be attributed to a defect early in thyroid gland embryogenesis, as the glandular tissue makes its way to a pre-tracheal position. The more distal the location of the ectopic tissue from typically neighboring structures, such as the recurrent laryngeal nerves or the superior thyroid artery, the greater the likelihood for issues such as hyperthyroidism, compression of nearby neurovascular structures, and malignancy. Depending on the exact anatomical location and hormonal activity of the tissue, management can range from administration of iodine to surgical resection. This report discusses a case of ETT discovered during the resection of an anterior mediastinal mass, possible treatment, and management plans.

## Introduction

Typically, the thyroid gland is found in the anterior neck between the second and fifth tracheal cartilages. However, variations in anatomy have been reported. Ninety percent of cases of ectopic thyroid tissue (ETT) are isolated to the lingual region [1,2]. ETT located in more distal areas is much less common. Mediastinal ETT is particularly rare, accounting for only 1% of all ETT cases [1,3].

ETT is a rare finding that arises due to aberrant migration of thyroid gland tissue during embryological development. It is most frequently found in the lingual region but can also be located in sublingual, pre-laryngeal, or mediastinal regions. The prevalence of ETT is higher in females than males, which is consistent with the higher incidence of thyroid diseases in women [4].

The clinical presentation of ETT varies depending on the anatomical location. Lingual ETT often presents as a midline mass at the base of the tongue, which may cause dysphagia, dysphonia, or respiratory distress if large enough [2]. Mediastinal ETT may present with symptoms related to compression of adjacent neurovascular structures, such as cough, dyspnea, chest pain, or superior vena cava syndrome. In some cases, ETT may be asymptomatic and discovered incidentally during imaging studies [1].

Suspicion for ETT may arise when a patient presents with a midline neck mass or symptoms of compression without a palpable thyroid gland in its normal anatomical position. Imaging studies such as ultrasound, CT scans, or MRI can help identify ETT. Functional studies like thyroid scintigraphy using technetium-99m or iodine-123 can confirm ETT by demonstrating uptake of the radiotracer. Fine-needle aspiration biopsy can provide cytological confirmation of thyroid tissue and help exclude malignancy [3].

## Case presentation

**Figure 1:**
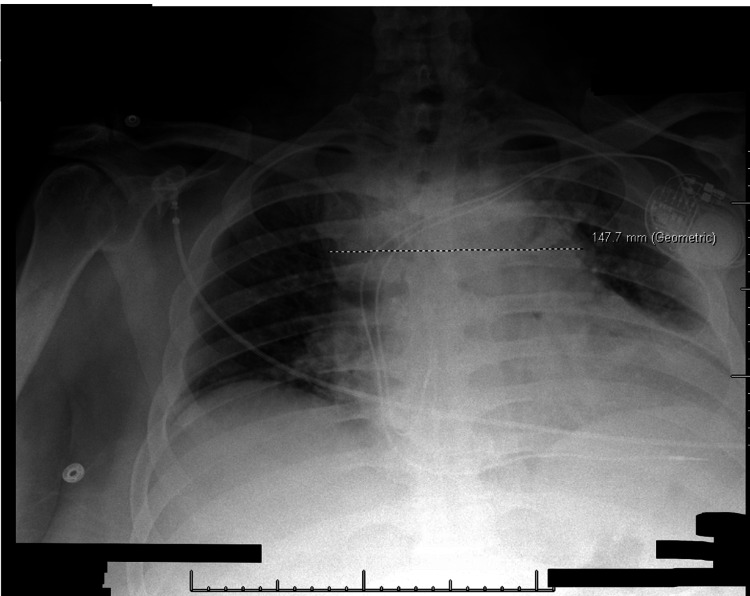
Chest 1 View AP/PA X-ray A frontal view of the chest was obtained. There is a stable appearance of a left chest pacemaker. The heart is mild to moderately enlarged with enlargement of the mediastinum measuring approximately 15 cm. There is mild pulmonary vascular congestion. Mild linear densities are seen at the bilateral lung bases which may represent atelectasis.

A 57-year-old male with a past medical history of insulin-controlled type 2 diabetes mellitus with nephropathy, stage 4 chronic kidney disease (CKD), atrial fibrillation, hypertension, and hypercalcemia secondary to hyperparathyroidism presented with bulky mediastinal adenopathy. The patient was managed and treated at the hospital and subsequently underwent a mediastinoscopy for evaluation of the anterior mediastinal mass, mediastinal adenopathy, and localized enlarged lymph nodes (Figure 1) found by chest X-ray. Ethics committee approval was obtained from the Wayne State University Institutional Review Board (Approval #: 2024 108) and patient consent was also obtained.

**Figure 2:**
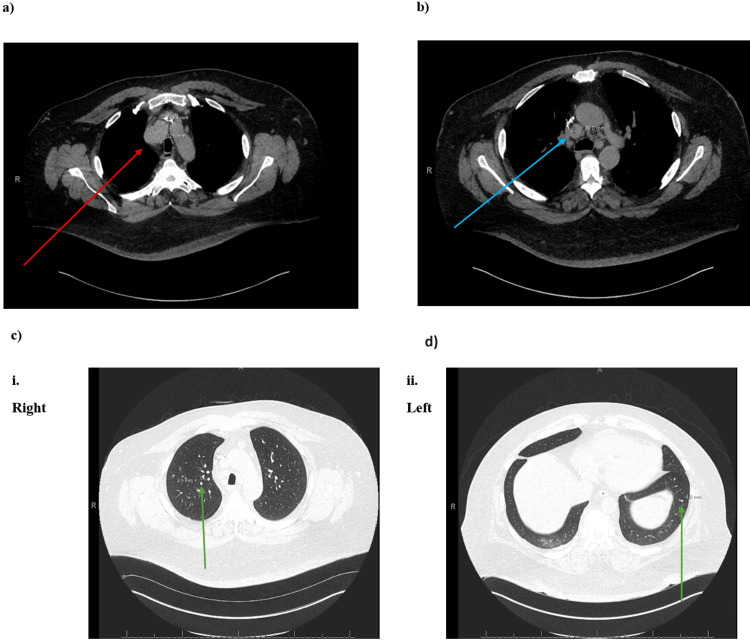
Chest CT Scan Mediastinal Findings: a) Stable mediastinal mass measuring 4.6 x 3.3 cm (indicated by red arrow). b) Stable mediastinal lymphadenopathy measuring up to 1.9 cm in the short axis (indicated by blue arrow). Lungs and Pleura Findings: c) Multiple stable pulmonary nodules throughout both lungs, measuring up to 4 mm. The presence of these nodules had been compared to previous scans, and there was no evidence of new nodules. The largest nodule on each side is indicated by a green arrow, confirming stability and no new areas of concern. Note: The arrows are digitally produced and highlight the areas of interest.

Incision of the platysma muscle and the pre-tracheal plane revealed dense fibrosis which was dissected and electrocauterized in the anterior pre-tracheal plane. Below the innominate artery, there was a large mass on the right paratracheal side that correlated with the mass seen in the CT scan (Figure 2). Dissection of this mass revealed a very high degree of vascularity. 

The patient subsequently underwent a biopsy of a mediastinal mass and a biopsy of a right paratracheal mass at station #2R. Initially, it was suspected that the findings could represent a granulomatous disease, such as sarcoidosis, or tumors in the mediastinum that had possibly metastasized to the lymph nodes in the area. Additionally, the presence of a thymoma was also considered but excluded after frozen pathology revealed that the mediastinal mass consisted of thyroid tissue and the right paratracheal mass was composed of thyroid tissue with dense fibrosis and granulomas.

The differential diagnosis for an anterior mediastinal mass includes thymomas, teratomas/germ cell tumors, lymphoma, and ETT. In this patient, a mediastinoscopy was performed, during which a pathologist immediately tested the tissue, revealing ETT with dense fibrosis and granulomas. This finding excluded other diagnoses such as thymomas and malignancies (refer to Table 1).

**Table 1:** Pathology Report Summary Examination of specimens A-C and F shows areas of frozen section artifact. Examination of specimens A-F shows no neutrophilic infiltrate, no chronic inflammatory cell infiltrate, no granulomas, and no carcinoma identified. Clinical correlation is suggested. All/1: indicates that out of all the lymph nodes examined, 1 node is positive for pathology. In this case, the pathology was the presence of ectopic thyroid tissue. No G: means "no grade" or "no grading," suggesting that no specific histological grade was assigned to the pathological findings in the sample because the grading system does not apply to the findings and the pathology did not warrant grading due to this not being cancerous.

Specimen Identifier	Body Region	Specimen Appearance and Measurements	Pathologist Interpretation
A	Right paratracheal mass	Red-tan rubbery soft tissue fragment measuring 0.5 cm in greatest dimension	Frozen section diagnosis: Thyroid tissue
B	Right paratracheal mass	2 red-tan soft tissue fragments measuring 0.4 and 0.8 cm in greatest dimension	Frozen section diagnosis: Thyroid tissue
C	Lower right paratracheal mass	Multiple red-tan rubbery soft tissue fragments aggregating to 0.8 x 0.5 x 0.2 cm	Frozen section diagnosis: Dense fibrosis inflammation, few giant cells
D	Lower right paratracheal mass	2 red-tan soft tissue fragments measuring 0.2 and 0.5 cm in greatest dimension	All/1, no G
E	2R lymph node	3 red-tan soft tissue fragments ranging from 0.2 to 0.6 cm in greatest dimension	All/1, no G
F	2R lymph node	Red-tan rubbery portion of soft tissue measuring 0.9 cm in greatest dimension	Frozen section diagnosis: Dense fibrosis inflammation, few giant cells

## Discussion

To confirm the diagnosis and rule out malignancy, a fine-needle aspiration is recommended. Additionally, thyroid function testing (TSH, T3, and T4 levels) is essential to determine if the patient has developed hypo- or hyperthyroidism. According to the American Thyroid Association guidelines [5], if the ETT becomes symptomatic, causes compression, or is suspicious of malignancy, surgical resection via thoracoscopic surgery is indicated. Post-surgery, thyroid hormone replacement therapy with Levothyroxine may be necessary if thyroid dysfunction is detected.

ETT in the mediastinum can present as benign nodules causing compression symptoms or as malignant tumors such as medullary or papillary thyroid carcinoma. Benign nodules may be asymptomatic or cause symptoms like superior vena cava syndrome, cough, stridor, and dysphagia [6]. Although rare, malignant transformation of ETT has been documented, particularly with papillary carcinomas in the glandular tissue [7].

Embryologically, the thyroid and parathyroid glands both develop from the branchial apparatus [8]. It is plausible that the ETT in this patient includes parathyroid tissue, contributing to hyperparathyroidism. Therefore, monitoring hormone levels (PTH, calcitonin, TSH, T4, and T3) is crucial.

This patient's secondary hyperparathyroidism is likely due to chronic kidney disease, but ectopic parathyroid tissue could exacerbate hypercalcemia, leading to complications such as kidney stones, abdominal pain, fatigue, osteoporosis, and skeletal frailty [9,10]. Conversely, excess calcitonin could reduce blood calcium levels, and excess TSH could induce hyperthyroidism symptoms like weight loss, heat intolerance, palpitations, tremors, and nervousness [11,12].

Follow-up includes regular thyroid function tests every six to twelve months and annual imaging (ultrasound or CT scans) to monitor the ETT. Regular clinical assessments are also necessary to detect neurovascular compression or thyroid dysfunction. Adhering to these guidelines, supported by endocrinology and oncology recommendations, should effectively manage the patient's condition and prevent complications.

## Conclusions

Going forward, a multidisciplinary approach will need to be employed in order to manage this patient. Endocrinology will have to provide an assessment and monitor the ETT and its impact on overall thyroid function. This includes regular thyroid function tests (TSH, T3, and T4 levels) and a fine-needle aspiration to exclude malignancy. If thyroid dysfunction is detected, thyroid hormone replacement therapy with levothyroxine will be necessary. Nephrology will have to focus on the patient's CKD and the potential exacerbation of hyperparathyroidism due to the ETT. They will need to frequently monitor renal function, calcium, and phosphate levels, and manage secondary hyperparathyroidism with phosphate binders, vitamin D analogs, and calcium analogs. Internal medicine will need to oversee the patient’s overall health, coordinate care among specialists, and conduct regular clinical assessments and physical exams to monitor for neurovascular compression or thyroid dysfunction symptoms. Thoracic surgery will need to be involved in evaluating the need for surgical resection of the ETT if it became symptomatic, caused compression, or was suspicious of malignancy, with the potential intervention being thoracoscopic surgery.

Alternative treatments include conservative management with regular monitoring and medical therapy for thyroid dysfunction. Radioiodine therapy will be considered for hyperfunctioning ETT. The potential resection of the ETT via thoracoscopic surgery will be prioritized for symptomatic or suspicious cases, while medical management will focus on controlling thyroid and parathyroid hormone levels to attenuate CKD impact. In similar cases, the chosen treatment approach will be tailored to the patient's symptoms, risk of malignancy, and the ETT’s impact on overall health. In conclusion, managing this patient and patients who present similarly requires a comprehensive multidisciplinary approach that integrates endocrinology, nephrology, internal medicine, and thoracic surgery with regular monitoring of thyroid function, renal function, and hormone levels. Such a plan can serve as a guide to manage similar cases in order to optimize patient health outcomes. 
